# Adrenergic signals influence proteomic responses in breast cancer cells

**DOI:** 10.3389/fnins.2025.1608017

**Published:** 2025-09-17

**Authors:** Manuel Carrasco, Ole Vidhammer Bjørnstad, Heidrun Vethe, Lars A. Akslen

**Affiliations:** ^1^Centre for Cancer Biomarkers CCBIO, Department of Clinical Medicine, Section for Pathology, University of Bergen, Bergen, Norway; ^2^Department of Pathology, Haukeland University Hospital, Bergen, Norway

**Keywords:** breast cancer, adrenergic signaling, noradrenaline, propranolol, proteomics

## Abstract

**Introduction:**

Breast cancer remains a major health challenge due to its molecular heterogeneity and complex interactions with the tumor microenvironment. Adrenergic signaling, mediated by stress hormones such as noradrenaline, has emerged as a potential regulator of cancer progression, influencing cell proliferation, cell adhesion, migration, and invasion.

**Methods:**

This study investigates the effects of adrenergic modulation on breast cancer spheroids from basal-like (MDA-MB-231, BT549) and luminal-like (T47D, MCF7) cell lines, using 3D culture systems as a more physiologically relevant model compared to traditional 2D monolayer cultures. The 3D spheroid model better recapitulates the structural complexity of tumors, providing insights into cell–cell and cell-matrix interactions that influence signaling pathways and drug responses.

**Results:**

Noradrenaline treatment significantly reduced spheroid size, invasion capacity, and the expression of EMT-related markers and integrins in MDA-MB-231 cells. These effects were partially reversed by propranolol, a non-selective beta-adrenergic receptor antagonist. Luminal-like spheroids, characterized by low ADRB2 abundance, displayed limited responsiveness to adrenergic modulation. Proteomic analysis revealed distinct subtype-specific responses, with basal-like spheroids showing pronounced alterations in pathways related to proliferation, cytoskeletal dynamics, epithelial-mesenchymal transition, and metabolism, whereas luminal-like spheroids exhibited minimal changes.

**Discussion:**

Our findings reveal heterogeneity in adrenergic receptor signaling across basal-like and luminal-like breast cancer cell lines, and also within the basal-like subgroup. This diversity underscores the complexity of adrenergic signaling in breast cancer and highlights the advantages of 3D culture systems. These results provide valuable insights into the subtype-specific patterns of response to adrenergic signaling that contribute to tumor progression and may inform future studies including evaluation of therapeutic strategies.

## Introduction

Breast cancer continues to be a significant global health concern, serving as the leading cause of cancer related mortality in women worldwide (Sung et al., 2021). The heterogeneous nature of these tumors is well known, with various molecular subtypes presenting different clinical characteristics, therapeutic responses, and prognostic outcomes (Yersal and Barutca, 2014). Over the last decade, integration of genomic, transcriptomic, and proteomic data has revealed a spectrum of involved pathways and corresponding molecular markers. This multi-omics approach has emphasized the importance of the breast cancer microenvironment (Nolan et al., 2023).

Recent developments in the emerging field of cancer neuroscience have indicated that the sympathetic nervous system, and in particular adrenergic signaling mechanisms, may play a role in breast cancer progression and response to therapy (Zahalka et al., 2017; Zahalka and Frenette, 2020). Chronic stress might significantly enhance adrenergic signaling by increasing noradrenaline turnover within the tumor microenvironment (Jayachandran et al., 2023; Eng et al., 2014). This localized effect is thought to be more impactful than circulating catecholamine levels, fostering tumor innervation and promoting cancer progression.(Zahalka and Frenette, 2020).

The sympathetic nervous system releases neurotransmitters like noradrenaline which binds to adrenergic receptors distributed throughout various tissues, including cancer cells (Karemaker, 2017). Studies have indicated that adrenergic signaling impacts tumor behavior, by inducing cell proliferation, migration, and invasion (Renz et al., 2018; Kim-Fuchs et al., 2014; Zhang et al., 2010). Beta-blockers such as propranolol, traditionally used to manage cardiovascular conditions (Wehrwein et al., 2016), have been shown to modulate adrenergic signaling pathways and are emerging as potential therapeutic agents in cancer treatment (Jansen et al., 2014; Barron et al., 2011; Watkins et al., 2015; Beg et al., 2018). The association between beta-blockers and breast cancer prognosis has been investigated, however with conflicting results, potentially reflecting the inherent differences between breast cancer subtypes (Barron et al., 2011; Melhem-Bertrandt et al., 2011; Sørensen et al., 2013; Cardwell et al., 2013; Spera et al., 2017; Modi et al., 2020; Lorona et al., 2021; Kim et al., 2017; Lofling et al., 2022; Scott et al., 2022; Scott et al., 2024). While some studies reported improved outcomes (Melhem-Bertrandt et al., 2011; Lofling et al., 2022), others have found no significant association or even suggested adverse effects (Scott et al., 2022; Scott et al., 2024). These inconsistencies emphasize the need for relevant model systems to elucidate subtype-specific effects and potential mechanisms of action.

To further address the questions regarding differential effects of adrenergic agonists and beta-blockers on specific molecular subtypes of breast cancer, we generated spheroids from four different BC cell lines: two basal-like (MDA-MB-231 and BT549) and two luminal-like (T47D and MCF7) cell lines. Breast cancer spheroids were treated with noradrenaline and propranolol in 3D culture, and the responses to adrenergic modulation were characterized by downstream proteomics responses, spheroid growth, invasion capacity, and morphological alterations. Our results indicate subtype-dependent adrenergic signaling responses in breast cancer cells by distinct proteomic profiles. Basal-like cell lines exhibited more pronounced changes, whereas luminal-like cell lines displayed minor changes.

Our findings contribute to a deeper understanding of the interplay between adrenergic signaling and intrinsic breast cancer biology. They highlight the heterogeneous responses to beta-blocker treatment across different tumor subgroups, which may explain the conflicting results in clinical studies.

## Results

### Noradrenaline treatment reduces spheroid growth and invasion capacity in basal-like breast cancer cells

**Figure 1:**
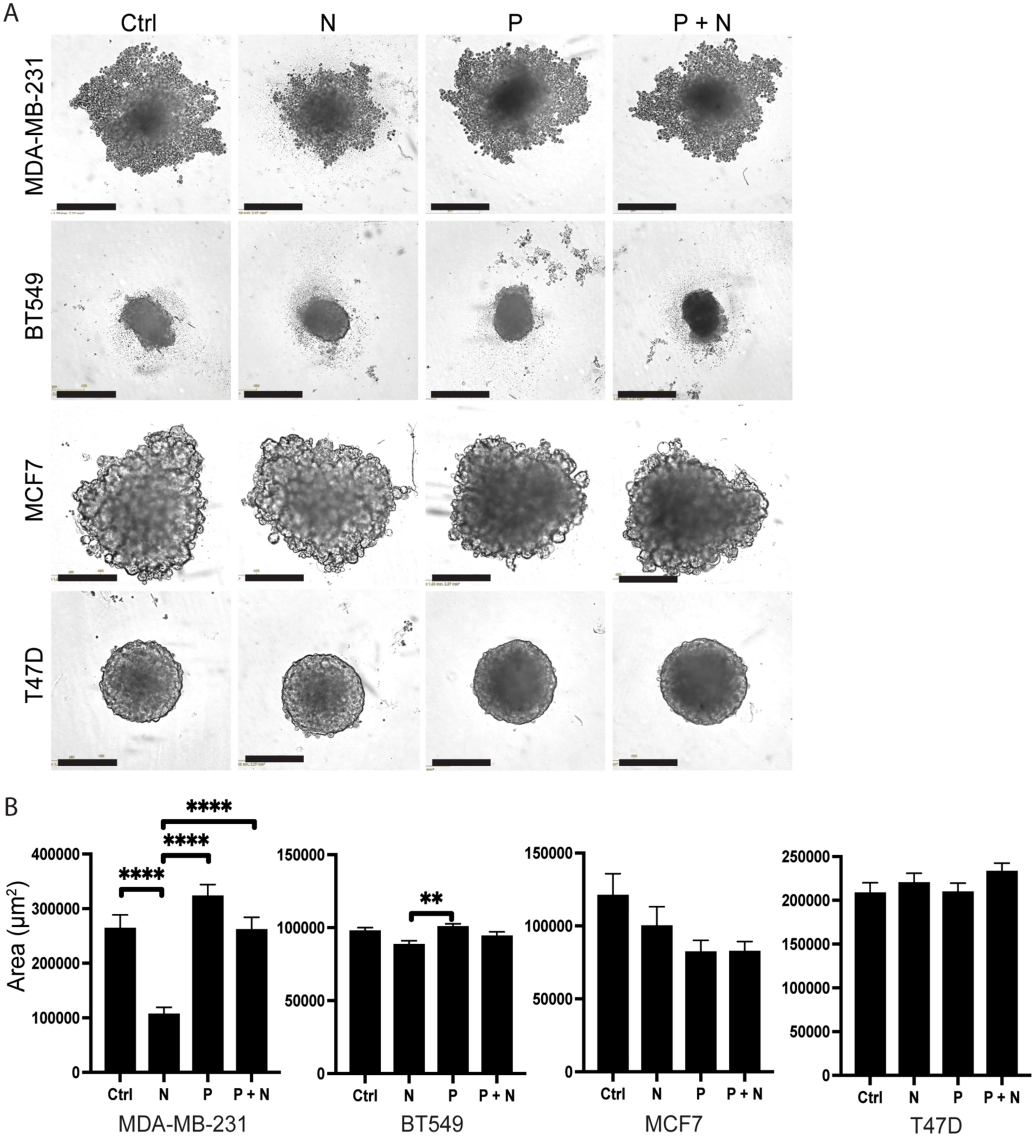
Noradrenaline alterations on spheroid area after 48 h treatment. **(A)** Representative microscope images of spheroids derived from MDA-MB-231, BT549, MCF7, and T47D cell lines. Spheroids were incubated for 48-h in culture medium containing either: culture medium (Ctrl), 10 μM noradrenaline (N), 10 μM propranolol (P), or a combination of 10 μM noradrenaline and 10 μM propranolol (N + P). Scale bar = 400 μm. **(B)** Quantitative analysis of spheroid areas using IncuCyte Analysis Software, with a sample size of *n* = 46 for each experimental condition. Error bars represent standard errors of the mean (SEM). ** *p* ≤ 0.01, **** *p* ≤ 0.001.

To investigate the effect of adrenergic signaling in BC models that better resemble the three-dimensional structure found *in vivo,* we generated spheroids from four different BC cell lines: two basal-like (MDA-MB-231 and BT549) and two luminal-like (T47D and MCF7) lines. The various BC cell lines demonstrated different spheroid morphologies *in vitro*, as reported previously (Kenny et al., 2007; Bjornstad et al., 2024), with differences also within the subtypes (Figure 1A). Six days after generation of spheroids, these were treated for 48 h with either 10 μM noradrenaline (N), 10 μM propranolol (P), or their combination (N + P), respectively. The choice of 10 μM of noradrenaline was justified based on its alignment with physiological levels observed in the tumor microenvironment, in accordance with previous literature (Zhang et al., 2020; Nilsson et al., 2007; Choi et al., 2015). Our pilot studies indicated its effectiveness within a non-toxic range. The spheroid area was quantified 48 h post-treatment, corresponding to the end of the experimental period, using IncuCyte Analysis Software (Figure 1B).

Noradrenaline treatment caused a 60% reduction in the average spheroid area in MDA-MB-231 cells compared to controls (Figures 1A,B). Interestingly, this effect was reversed when noradrenaline and propranolol were simultaneously added to the culture medium, suggesting that spheroid size reduction was mediated by beta-adrenergic receptor signaling. BT549 spheroids displayed an area decrease of 9.5% when treated with noradrenaline compared to controls (not significant). In contrast, luminal-type spheroids showed no significant change in average size under these treatment conditions.

In contrast, MCF7 spheroids displayed a modest, non-significant reduction in area following propranolol treatment alone. This may reflect a cell line-specific response independent of noradrenaline signaling (Figures 1A,B). To investigate the limited response of luminal-like spheroids, we assessed ADRB2 expression. Immuno-histochemistry showed strong staining in basal-like spheroids, but minimal in MCF7 and T47D (Supplementary Figures 1A–D). Quantification of ADRB2-positive cells confirmed these differences (Supplementary Figure 1E), and this was consistent with CCLE RNA-seq data showing low ADRB2 mRNA levels in luminal-like lines (Supplementary Figure 1F).

**Figure 2:**
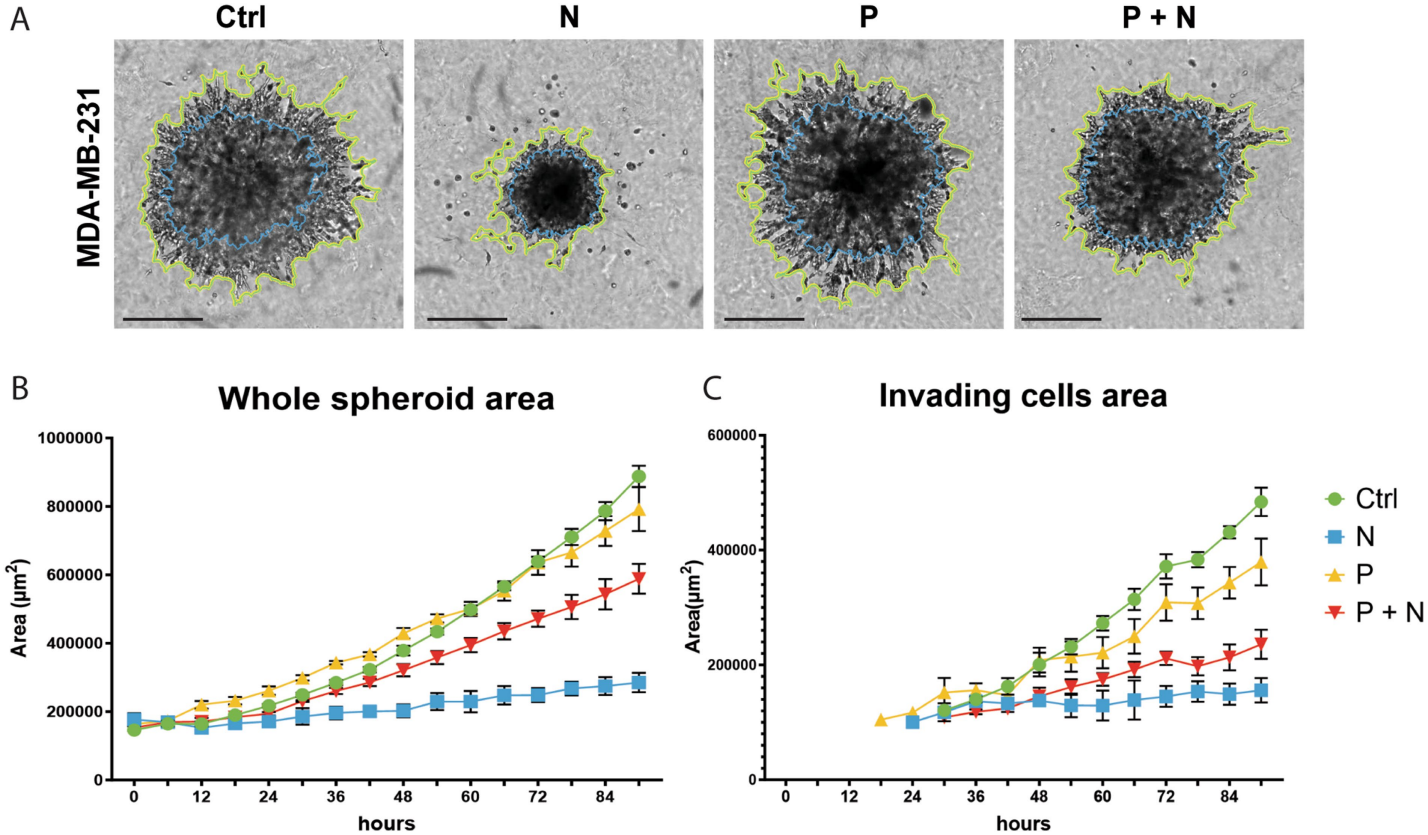
Invasion assay of MDA-MB-231 cells under adrenergic stimulation. **(A)** Representative images of spheroids after treatment: control (Ctrl), 10 μM noradrenaline (N), 10 μM propranolol (P), or their combination (P + N). The yellow line marks the spheroid area, while the blue line highlights the invading cell area. **(B)** Quantification of the whole spheroid area over time. **(C)** Quantification of the invading cell area over time. Noradrenaline significantly reduced the invasion capacity of MDA-MB-231 cells compared to control. Co-treatment with propranolol partially restored the invasion capacity (P + N group). Statistical analysis shows significant differences between groups. Error bars represent standard errors of the mean (SEM). AUC values and 95% confidence intervals for each condition can be found in Supplementary Table 1.

To further investigate the functional consequences of adrenergic signaling, we conducted a spheroid invasion assay (Figure 2). MDA-MB-231 spheroids treated with noradrenaline showed a significant reduction in invasive capacity, with a 46% reduction in AUC for whole spheroid area and a 40% reduction in AUC for invading cell area compared to untreated controls (*p* < 0.001, Welch’s t-test). Interestingly, this reduction was partially reversed by propranolol, suggesting the involvement of beta-adrenergic receptor signaling in regulating invasion (Supplementary Table 1 for 95% confidence intervals).

Proteomic analysis revealed downregulation of VIM and STMN1, proteins involved in cytoskeletal remodeling and microtubule destabilization. These findings were supported by broader changes in additional EMT-related proteins, including cadherins, various integrin subunits, MMPs, and other adhesion molecules, as observed in noradrenaline-treated spheroids (Supplementary Figure 2). Together, these data suggest that adrenergic stimulation impairs the invasive potential of MDA-MB-231 spheroids. No cell invasion was observed for BT549 cells under our experimental conditions. Luminal-like spheroids (T47D and MCF7) revealed minimal invasive behavior in 3D models (Kenny et al., 2007; Manuel Iglesias et al., 2013).

**Figure 3:**
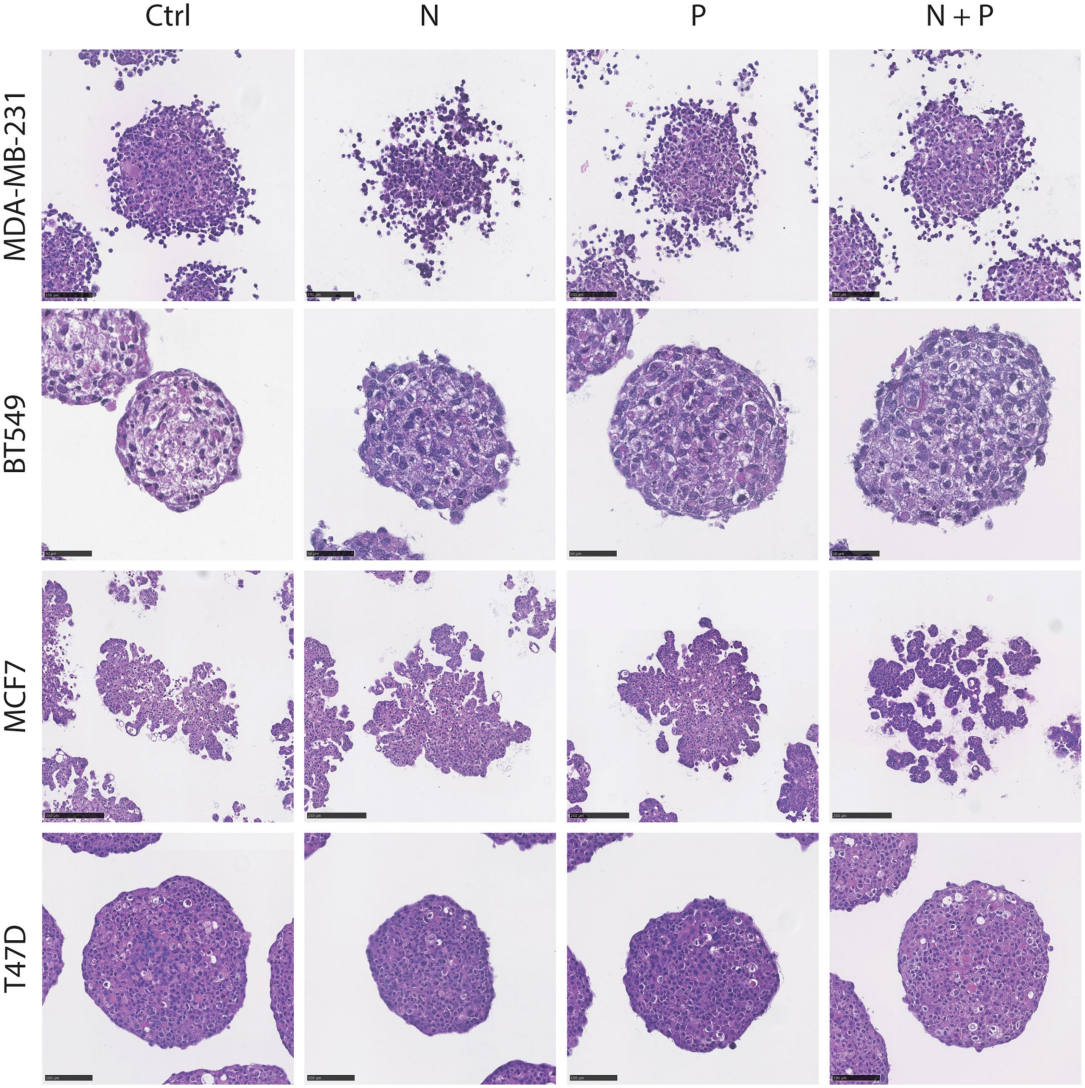
Hematoxylin and Eosin (H&E) staining of spheroids post-treatment. Representative images of spheroids from four cell lines (MDA-MB-231, BT549, T47D and MCF7) after 48 h-treatment with noradrenaline (N), propranolol (P) or a combination of both (N + P). Scale bar: BT549, 50 μm; MDA-MB-231 and T47D, 100 μm; MCF7, 250 μm.

Hematoxylin and eosin (H&E) staining is shown in Figure 3, with different morphologies observed between the spheroid models, including varying degrees of cell adhesion, and with increased dys-cohesion in MDA-MB-231 cells after noradrenaline treatment.

### Global proteome analysis reveals different responses in basal-like and luminal-like breast cancer spheroids to noradrenaline and propranolol treatment

**Figure 4:**
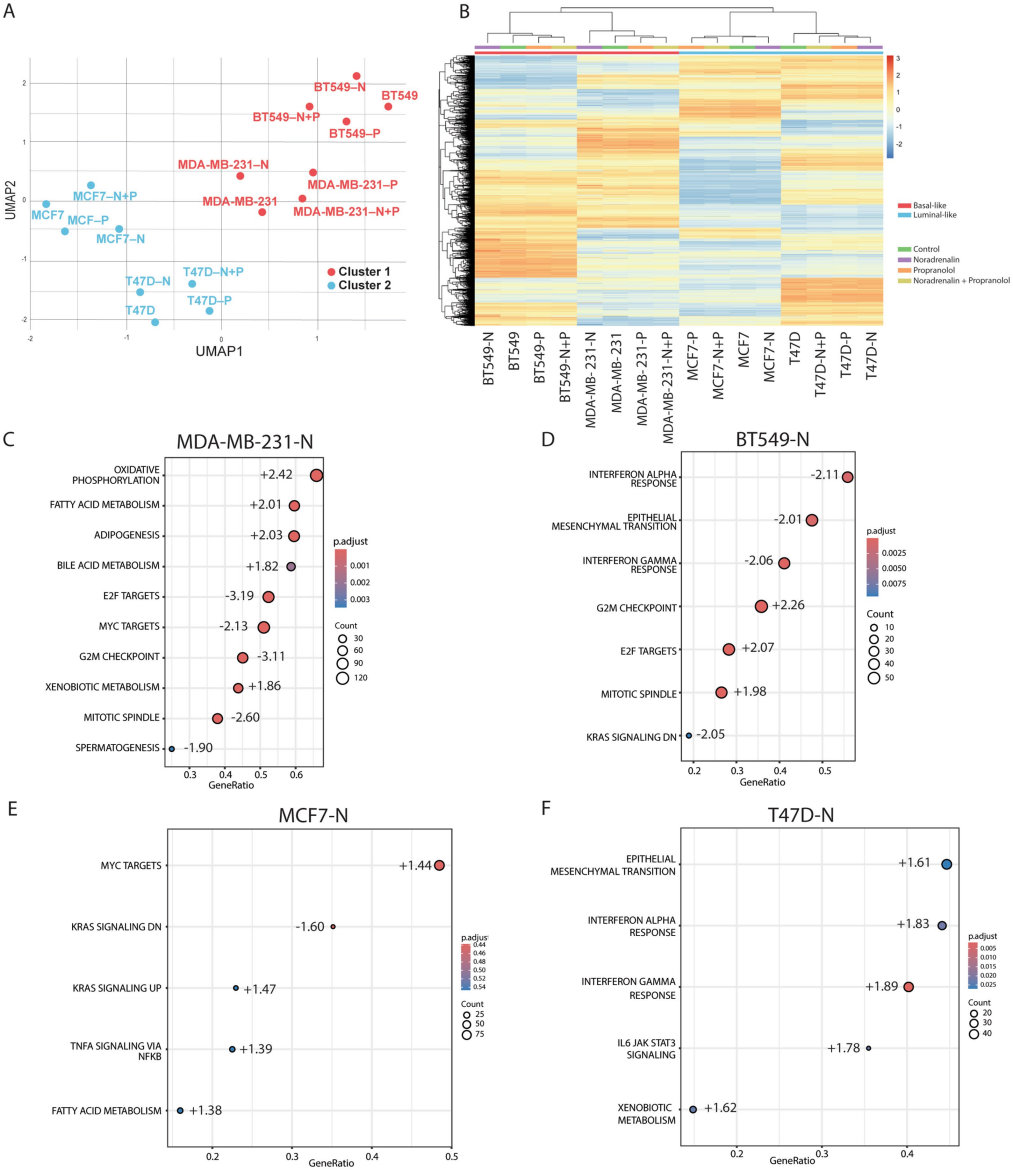
Comprehensive proteomic analysis of spheroids generated from four breast cancer cell lines subjected to adrenergic modulation. **(A)** UMAP (Uniform Manifold Approximation and Projection) visualization illustrating the full proteome of four cell lines (*n* = 2 for each cell line and condition) MDA-MB-231, BT549, MCF7, and T47D, before and after treatment with 10 μM noradrenaline (N), 10 μM propranolol (P), and a combination of noradrenaline + propranolol (N + P). The UMAP reflects two clusters, which are identified with the basal-like cell lines (cluster 1, in red) and luminal-like (cluster 2, in blue) **(B)** Hierarchical clustering of the entire proteome across the four cell lines under different treatment conditions. The color-coded bar represents z-scored expression values: blue signifies downregulation, and red indicates upregulation. In basal-like cells, a greater degree of hierarchical separation is seen in the cells treated with N with respect to the rest of the treatments than in the spheroids derived from luminal-like cells. **(C–F)** GSEA enrichment analysis for **(C)** MDA-MB-231-N, **(D)** BT549-N, **(E)** MCF7-N and **(F)** T47D-N compared to control utilizing the hallmark gene set collection (Subramanian et al., 2005). Enriched gene sets are displayed with an upper limit of 10 gene sets displayed. GeneRatio refers to the fraction of genes from the cell lines represented in the utilized gene sets. Normalized enrichment scores, an evaluation of whether genes are over- or underrepresented at either end of the ranked list of genes, are also displayed by the dots in the plot. Count refers to the dot size which displays the total number of proteins counted for each gene set. *p*-value of each gene set is displayed in gradient colors. *p* ≤ 0.05 for panel **(C,D,F)** and the two first gene sets of panel **(E)**.

Mass spectrometry-based proteomics analysis of breast cancer cell lines MDA-MB-231, BT549, T47D and MCF7 before and after adrenergic modulation quantified a total of 7,770 proteins. Uniform Manifold Approximation and Projection (UMAP) plotting revealed distinct clustering of basal-like (MDA-MB-231, BT549) and luminal-like (T47D, MCF7) subtypes (Figure 4A), independent of the treatment (noradrenaline, propranolol). However, the individual treatment conditions induced distinct proteomic profiles, as confirmed by hierarchical clustering (Figure 4B). Specifically, basal-like spheroids (MDA-MB-231-N and BT549-N) showed a marked proteomic change in response to noradrenaline compared to both alternative treatments (propranolol alone or in combination with noradrenaline). In contrast, luminal-like spheroids treated with noradrenaline (MCF7-N and T47D-N) showed a more restrained proteomic shift. This suggests that adrenergic signaling has a stronger impact on the proteomic profile of basal-like breast cancer spheroids than in luminal-like ones.

Propranolol treatment changed the protein expression patterns in spheroids from BC cell lines, indicating *β*-adrenergic receptor involvement. Interestingly, combination of noradrenaline and propranolol did not fully rescue the proteomic alterations of noradrenaline treatment alone but led to a separate alteration of the proteomic profiles (Figure 4A). This suggests that noradrenaline signaling activation is not restricted to beta-receptors activation.

### Adrenergic stimulation influences cellular pathways at the proteomic level

We then analyzed, at the global protein level, specific biological hallmark gene sets from the MSigDB database (Liberzon et al., 2015) (Figures 4C–F).

In MDA-MB-231 spheroids treated with noradrenaline, we observed an enrichment of metabolic processes including oxidative phosphorylation, fatty acid metabolism, and increased adipogenesis, suggesting a metabolic reprogramming towards energy production and lipid processing, potentially as an adaptive response to adrenergic signaling. In parallel, we observed downregulation of cell cycle-associated pathways (E2F and MYC targets, G2/M checkpoint activity, and mitotic spindle-associated proteins), suggesting a reduction in proliferation signaling, which may reflect a shift towards stress adaptation or altered cellular priorities (Figure 4C; Supplementary Figure 3, displaying the GSEA plots). These findings align with the observed decrease in Ki67 and the overall reduced spheroid size.

In noradrenaline-treated BT549 spheroids (BT549-N), we observed increased G2/M checkpoint activity, E2F targets, and mitotic spindle-associated factors, suggesting enhanced cell cycle progression. In contrast to MDA-MB-231, metabolic reprogramming was less prominent. Instead, interferon-alpha and gamma responses, as well as pathways associated with epithelial-mesenchymal transition (EMT), were notably downregulated (Figure 4D; Supplementary Figure 3). This pattern suggests a shift towards enhanced cell cycle progression and proliferation.

For luminal-like MCF7-N spheroids, pathway activation for both MYC and KRAS was increased (Figure 4E; Supplementary Figure 4) but the overall proteomic shift was modest compared to basal-like cells, possibly due to low ADRB2 expression as shown by IHC and mRNA data (Supplementary Figure 1) and/or the presence of compensatory signaling pathways that could buffer downstream effects.

In T47D-N spheroids, we observed enrichment for EMT, interferon-alpha and gamma responses, as well as IL-6/JAK/STAT3 signaling (Figure 4F; Supplementary Figure 4, displaying the GSEA plots). This suggests a tendency toward cellular dedifferentiation, amplified immune signaling, and activation of inflammation pathways, without major alterations in cell cycle programs.

Taken together, adrenergic stimulation induced subtype-specific responses: basal-like cell lines showed contrasting proliferation patterns, with MDA-MB-231 favoring metabolic reprogramming and BT549 increasing proliferation, while luminal-like cell lines exhibited no significant changes in proliferation-related gene sets. This highlights the context-dependent effects of adrenergic signaling in breast cancer, potentially influenced by subtype-specific differences in receptor expression and downstream signaling pathways.

### Proteomic signatures and predicted pathways in breast cancer cells following noradrenaline treatment

Significantly differentially expressed proteins with *p* < 0.05 (*n* = 991 for MDA-MB-231, *n* = 355 for BT549, *n* = 368 for T47D, and *n* = 189 for MCF7) were identified by comparing noradrenaline-treated spheroids to controls. Ingenuity Pathway Analysis (IPA) revealed distinct effects of adrenergic signaling on breast cancer spheroids across basal-like (MDA-MB-231, BT549) and luminal-like (T47D, MCF7) subtypes.

In MDA-MB-231 spheroids, pathways related to cell cycle and genomic stability were inferred inhibited (negative z-score). Gene expression and translation pathways were also downregulated, alongside significant inhibition of apoptotic signaling. Cytoskeletal dynamics and cell migration pathways, including RHO GTPase signaling, were suppressed. In addition, metabolic pathway analysis revealed activation of fatty acid *β*-oxidation, suggesting a metabolic reprogramming towards lipid utilization under adrenergic stimulation.

In contrast, BT549 spheroids displayed activation of cell cycle and genomic stability pathways. Gene expression and translation pathways were also upregulated, with mild activation of apoptotic signaling. Cytoskeletal and migration-related pathways, including RHO GTPase signaling, were activated as well. Metabolically, BT549 showed strong activation of oxidative phosphorylation and sirtuin signaling, consistent with increased energy production and stress adaptation.

**Figure 5:**
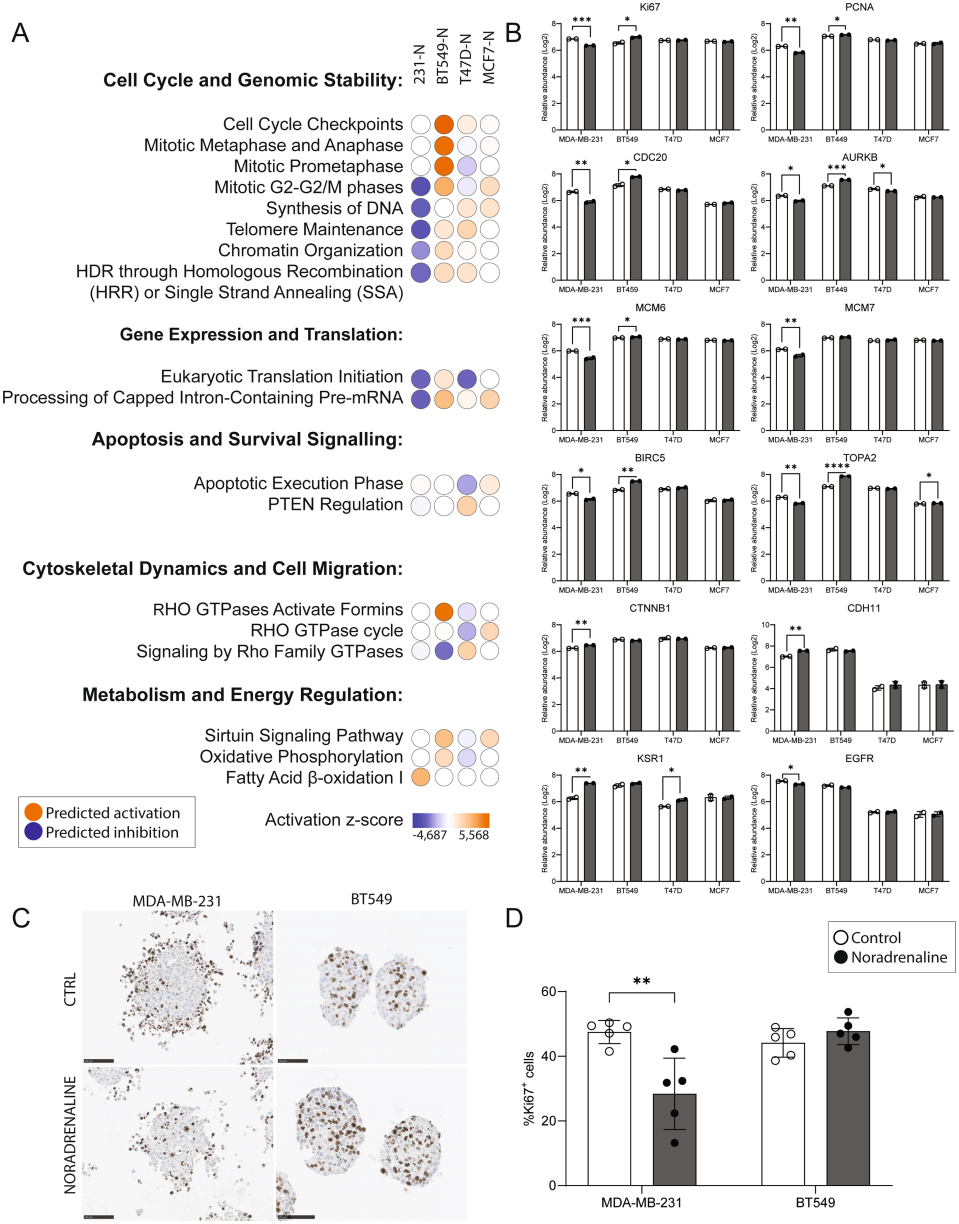
Impact of noradrenaline treatment on pathways, protein expression, and Ki67 staining in breast cancer spheroids. **(A)** Ingenuity Pathway Analysis (IPA) z-scores predicting activation or inhibition of key cellular processes across spheroids treated with 10 μM noradrenaline (N) for 48 h. Subtype-specific responses were observed in basal-like (MDA-MB-231, BT549) and luminal-like (T47D, MCF7) spheroids. Pathways related to cell cycle and genomic stability were predicted to be inhibited in MDA-MB-231 but activated in BT549, while luminal subtypes showed minimal regulation. Gene expression and translation pathways were inhibited in MDA-MB-231 and activated in BT549. Apoptotic pathways were inhibited in MDA-MB-231 but mildly activated in BT549. Cytoskeletal dynamics and cell migration pathways were inhibited in MDA-MB-231 and activated in BT549, with negligible effects in luminal subtypes. Metabolic pathways, including fatty acid β-oxidation and oxidative phosphorylation, were activated in both basal-like subtypes. **(B)** Log2 protein abundance changes in proliferation markers (KI67, PCNA, CDC20, AURKB), replication factors (MCM6, MCM7), and key signaling proteins (BIRC5, TOP2A, EGFR, KSR1) across spheroids following noradrenaline treatment. MDA-MB-231 exhibited significant downregulation of proliferation-related markers, while BT549 showed upregulation of the same proteins. Luminal subtypes displayed limited proteomic changes. **(C)** Representative images of Ki67 immunohistochemical staining in spheroids from basal-like MDA-MB-231 and BT549 cell lines, with and without noradrenaline treatment. Scale bars, 100 μm. **(D)** Quantification of Ki67-positive cells in MDA-MB-231 and BT549 spheroids. Noradrenaline treatment significantly reduced the percentage of Ki67-positive cells in MDA-MB-231 (*p* = 0.0062) and showed a trend of increased Ki67 positivity in BT549, though not statistically significant. Protein levels from two experiments are shown as means ± SD. *p ≤ 0.05, **p ≤ 0.01, ***p ≤ 0.005.

Luminal-like spheroids (T47D and MCF7) exhibited near-neutral activity scores across most pathways, indicating a reduced or heterogeneous response to noradrenaline treatment. Gene expression, apoptosis, cytoskeletal organization, and migration-related pathways showed only modest or inconsistent changes. Metabolic responses were limited, with MCF7 remaining largely unaffected and only mild pathway activation observed in T47D (Figure 5A).

Protein level analyses confirmed the pathway-level predictions (Figure 5B). In MDA-MB-231 spheroids, proliferation and replication markers, including KI67, PCNA, CDC20, AURKB, MCM6, MCM7, BIRC5, TOP2A, and EGFR, were significantly downregulated following noradrenaline treatment (*p* < 0.05). In contrast, BT549 spheroids exhibited significant upregulation of these same proteins (excluding EGFR), consistent with the activation of cell cycle pathways. In luminal-like spheroids, the proteomic responses were more limited. T47D spheroids showed significant downregulation of AURKB and upregulation of KSR1. In MCF7 spheroids, only a modest upregulation of TOP2A was observed, with no significant changes in other proteins.

Finally, the percentage of proliferative cells in basal-like spheroids was quantified, defined as cells showing positive staining for the Ki67 antibody, a well-established marker of cell proliferation (Figures 5C, D). Spheroids from the MDA-MB-231 cell line treated with 10 μM noradrenaline exhibited a significant reduction (40.2%) in the percentage of Ki67-positive cells compared to the control group (*p* = 0.0062). In contrast, spheroids from the BT549 cell line showed a trend towards a slightly increased percentage of Ki67-positive cells following noradrenaline treatment (8.1%), although this change was not statistically significant. These findings support the predictions from IPA regarding proliferation and align with the protein expression levels observed through mass spectrometry.

Taken together, IPA and protein level analyses highlight the subtype-specific responses of breast cancer spheroids to adrenergic signaling. Basal-like spheroids demonstrated pronounced and opposing effects: MDA-MB-231 spheroids showed downregulation of proliferation and cell cycle pathways alongside metabolic reprogramming, while BT549 spheroids exhibited enhanced proliferation and cell cycle progression. In contrast, luminal-like spheroids displayed a more muted proteomic response to noradrenaline, with only modest changes observed in key pathways and markers. These results underscore the heterogeneity of adrenergic signaling effects across breast cancer subtypes and suggest that the context-dependent modulation of cellular processes, such as proliferation, metabolism, and migration, may have implications for therapeutic targeting of adrenergic pathways in breast cancer.

## Discussion

This study investigated how adrenergic signaling influences breast cancer spheroids in a 3D model that mimics *in vivo* architecture (Kenny et al., 2007; Saraiva et al., 2020; Carrasco et al., 2022; Jin et al., 2023). We examined spheroid growth, invasive capacity, and the global proteomic response of basal-like (MDA-MB-231 and BT549) and luminal-like (T47D and MCF7) cell lines after treatment with noradrenaline and the beta-adrenergic receptor antagonist propranolol.

A significant finding in our study revolves around the differential responses of basal-like and luminal-like breast cancer spheroids to noradrenaline and propranolol. Specifically, noradrenaline treatment significantly reduced both the size of basal-like spheroids in the MDA-MB-231 model, as well as the invasive capacity. The observed size reduction was counteracted by blocking the beta-adrenergic receptors with propranolol, suggesting that adrenergic stimulation through beta-adrenergic receptors reduces the growth of this cancer cell line. In contrast, previous 2D data (Jin et al., 2023; Ouyang et al., 2019) showed that noradrenaline treatment induced higher proliferation in most basal-like cell lines, except for HS578T.

In our study, the size reduction observed after noradrenaline treatment of MDA-MB-231 cells was parallelled by functional data, proteomic information and pathway analyses. Expression of the proliferation marker Ki67 was significantly reduced, and IPA predicted downregulation of cell cycle and proliferation pathways, which aligned with the reduced expression of proliferation markers such as PCNA and CDC20. Taken together, these findings suggest a coordinated response to adrenergic stimulation that is consistent with reduced proliferative activity.

The therapeutic potential of beta-blockers, such as propranolol, has been extensively explored in breast cancer, with mixed results likely reflecting differences in adrenergic receptor expression, downstream signaling, and tumor microenvironmental context within and across tumor subtypes. Whereas some clinical evidence suggests that basal-like or triple-negative breast cancer, characterized by higher adrenergic receptor activity, may respond more favorably to beta-blocker therapy compared to luminal-like subtypes (Kim et al., 2017), this was not supported by our findings using the 3D MDA-MB-231 spheroid model. More studies are therefore needed to clarify this, and to separate between direct effects on tumor cells, and indirect influences via other cells and components in the intact *in vivo* tumor microenvironment. Notably, in a recent study, salbutamol, a selective agonist for β2-adrenergic receptors, was repurposed as an anti-cancer drug for triple negative breast cancer cell models (Jabloñski et al., 2023; Rivero et al., 2017). Salbutamol, in combination with paclitaxel, decreased proliferation in the MDA-MB-231 breast cancer cell line and inhibited tumor growth in a mouse model. These findings are in line with our present data on MDA-MB-231 cells and support the validity of our spheroid model.

Notably, previous studies using 2D monolayer cultures have reported somewhat contrasting findings. Ouyang et al. studied MCF7 (luminal-like) and MDA-MB-231 (basal-like) cells cultured as monolayers on plastic, reporting enhanced proliferation, invasion and migration upon noradrenaline stimulation (Ouyang et al., 2019). Similarly, another study on basal-like breast cancer cell lines (MDA-MB-231, MDA-MB-468, and BT549) using 2D cultures indicated that noradrenaline treatment and *β*2-adrenergic signaling promoted cell proliferation and neural growth factor production for most cell lines studied (Jin et al., 2023).

The differences between previous 2D studies and our current findings in 3D spheroids likely reflect the context-dependent nature of adrenergic signaling. Unlike 2D monolayers, 3D models better recapitulate *in vivo* conditions, including cell–cell and cell-matrix interactions, mechanical constraints, and gradients of nutrients and oxygen. These factors can modulate receptor availability, signaling intensity, and downstream biological responses. Our results therefore highlight the importance of using physiologically relevant 3D models to capture the complex behavior of breast cancer cells under adrenergic stimulation.

Proteomic responses after noradrenaline treatment revealed subtype-specific differences, with variation across cell lines. Differences were predicted in proliferation, metabolism, genome stability, and migration. In basal-like spheroids, MDA-MB-231 cells indicated inhibition of pathways regulating cell cycle, DNA repair, and cytoskeletal dynamics after adrenergic signaling, aligning with the observed downregulation of key markers such as Ki67, PCNA, and CDC20. In addition, we observed a deregulation of cytoskeletal markers (VIM and STMN1), integrin subunits, MMP16, and other cell adhesion molecules, all of which are functionally associated with invasion (Yue et al., 2012; Winter et al., 2021). This suggests that adrenergic signaling exerts anti-proliferative effects in this aggressive subtype. Interestingly, the divergent responses of MDA-MB-231 and BT549, despite their shared basal-like classification, may possibly reflect differences in *β*-adrenergic receptor subtype expression (e.g., β1 vs. β2), downstream signaling, or intrinsic metabolic flexibility. Receptor-independent influences might also be involved (Overman et al., 2019; Pasquier et al., 2011; Gargiulo et al., 2020). While MDA-MB-231 favored lipid oxidation and suppression of proliferation, BT549 showed enhanced oxidative phosphorylation and cell cycle activation, pointing to distinct adaptive strategies to adrenergic stimulation.

In this study, the response of luminal-like spheroids to adrenergic stimulation was less pronounced, showing no significant size change and fewer significantly regulated proteins following adrenergic signaling modulation, suggesting that luminal-like breast cancer cells may exhibit lower sensitivity to adrenergic signaling. The limited impact of noradrenaline treatment in luminal-like spheroids underscores the heterogeneity of adrenergic signaling effects. While T47D showed slight changes, including downregulation of AURKB and upregulation of KSR1, MCF7 displayed only a modest upregulation of TOP2A. These findings suggest that luminal subtypes may be less responsive to adrenergic signaling, potentially due to differences in receptor expression, as supported by minimal ADRB2 immunoreactivity observed in the luminal-like spheroids (MCF7, T47D), as well as the reduced ADRB2 levels (mRNA) found in luminal cells from the CCLE collection.

Metabolic pathway alterations, particularly fatty acid *β*-oxidation in MDA-MB-231 and oxidative phosphorylation in BT549, reflect some differences in the metabolic reprogramming induced by adrenergic signaling. This might suggest subtype-specific adaptations to stress or changes in energy demand. The activation of sirtuin signaling in BT549 supports this hypothesis, as it is often associated with enhanced metabolic and survival pathways (Carracedo et al., 2013; German and Haigis, 2015).

In our study, we employed a concentration of 10 μM noradrenaline to stimulate adrenergic signaling in breast cancer spheroids. This concentration is consistent with previous research investigating the effects of adrenergic stimulation on breast epithelial cells. For instance, Silva et al. (2024) utilized 10 μM concentrations of adrenergic agonists to study their impact on MCF10A non-tumorigenic breast epithelial cells, observing significant effects on cell adhesion, proliferation, and migration. Similarly, Oliveira et al. employed 10 μM concentrations of adrenergic agonists in their exploration of catecholamine synthesis and its influence on cell viability and colony formation in MCF10A and MCF7 breast epithelial cells (Amaro et al., 2020). These studies demonstrate that a 10 μM concentration of noradrenaline is effective for eliciting measurable responses in breast epithelial cells. Still, we acknowledge that a dose–response design could provide additional mechanistic insight and should be considered in future studies.

While our current study focused on non-selective β-adrenergic receptor modulation using propranolol, we agree that exploring *α*-adrenergic antagonists, as well as β1- and β2-selective blockers, would be valuable to further dissect receptor-specific effects in breast cancer spheroids. Of further importance, not all observed effects may be strictly mediated by canonical β-adrenergic receptor signaling (Overman et al., 2019; Gargiulo et al., 2020). Both noradrenaline and propranolol can exert non-receptor-mediated actions, including membrane interactions and inverse agonism (Baker et al., 2011), which may have contributed to the proteomic and phenotypic changes observed in our 3D cell models. These possibilities should be considered when interpreting the results.

Taken together, our 3D cell culture study shows subtype-specific variations in growth, invasion and proteomic responses to noradrenaline. Further studies are needed to understand the underlying mechanisms and assess how well this model translates to clinical applications in breast cancer. While our study provides insights into the effects of adrenergic signaling on some breast cancer spheroids, it is limited by its reliance on 3D *in vitro* models. Although spheroids are physiologically relevant and mimic some aspects of *in vivo* tumor structure, better than 2D *in vitro* models, they cannot fully replicate the complexity of the tumor microenvironment *in vivo*, including immune cell interactions and the complex stromal components. These limitations highlight the need for complementary approaches using more advanced systems, such as animal models, organoids and patient-derived xenografts and a broader assessment of adrenergic signaling across genetically diverse breast cancer models. Future work should consider the diversity in adrenergic receptor expression, within and across breast cancer subtypes, and the implications for therapy stratification.

### Methods

#### Tumor cell spheroid generation and drug treatment

Two basal-like (MDA-MB-231 and BT549) and two luminal-like (T47D and MCF7) cell lines were dissociated with 0.25% trypsin. 5,000 cells were seeded per well in Ultra-Low Cluster 96 well plate (Costar #7007, Corning) in 200 μL of medium. Medium was refreshed every other day. On day 6, noradrenaline tartrate (Sigma, N1100000) and/or propranolol hydrochloride (Sigma, P8688) was added to a final concentration of 10 μM. Previous studies suggested that in the tumor microenvironment, noradrenaline concentrations may reach as high as 10 μM (Zhang et al., 2020; Nilsson et al., 2007; Choi et al., 2015). Spheroids were collected for protein extraction and histology 48 h after treatment. All cells used tested negative for mycoplasma contamination using MycoAlert Mycoplasm Detection Kit (Lonza, LT07-318).

The TP53 mutational status of the four breast cancer cell lines (MDA-MB-231, BT549, MCF7 and T47D) was gathered from Leroy et al. (2014), with MDA-MB-231 exhibiting a mutation at nucleotide position c.839G > A, resulting in an amino acid substitution at p. R280K. BT549 cells harbored a c.747G > C mutation, leading to a p. R249S amino acid change. MCF7 cells were found to be wild type with respect to the TP53 gene. Lastly, T47D cells displayed a c.580C > T mutation, corresponding to a p. L194F substitution in the TP53 protein.

#### Basic morphology

Spheroids were fixated in 10% formalin for 15 min, washed with PBS twice, dehydrated and paraffin embedded. Sections of 4 μm were subsequently deparaffinized and rehydrated using xylene and graded ethanol washes, respectively. H&E staining was then performed using standard procedures.

#### Immunohistochemistry

Sections of 4 μm were deparaffinized and rehydrated using xylene and graded ethanol washes. The following primary antibodies were used at the indicated dilutions: mouse anti-Ki67 (Agilent M7240, 1:250), rabbit anti-beta 2 Adrenergic Receptor (ADRB2) (Abcam ab182136, 1:100). Immunoperoxidase staining was performed using EnVision (Dako) with diaminobenzidine (DAB) as the peroxidase substrate. Counterstaining with hematoxylin was performed to visualize nuclei.

### Proteomics analysis

#### Cell lysis, protein digestion, and processing

Cells were lysed in 4% SDS in 100 mM Tris-HCl (pH 8.5) and sonicated in a water bath for 30 s three times. Extracted proteins (10 μg per sample) were reduced with Tris(2-carboxyethyl)-phosphine and alkylated with chloroacetamide. SP3 (Single-Pot Solid-Phase-enhanced Sample Preparation) was used for protein capture, cleaning, and digestion, followed by desalting using C18 columns (SPE).

Tryptic peptides were dissolved in 100 mM triethyl ammonium bicarbonate and labeled with the TMT 18plex kit (ThermoFisher Scientific), using 16 out of 18 channels. The samples were fractionated into 8 fractions using the Pierce High pH Reversed-Phase Peptide Fractionation Kit (ThermoFisher Scientific) before LC-MS/MS analysis.

#### LC-MS/MS analysis

Approximately 0.5 μg of tryptic peptides (dissolved in 2% acetonitrile, 0.5% formic acid) were injected into an Ultimate 3,000 RSLC system (Thermo Scientific) coupled to an Orbitrap Eclipse mass spectrometer (Thermo Scientific) with an EASY-spray nano-electrospray ion source.

#### Trapping and desalting

The sample was loaded and desalted on a pre-column (Acclaim PepMap 100, 2 cm x 75 μm ID nanoViper column, packed with 3 μm C18 beads) at a flow rate of 5 μL/min for 5 min with 0.1% trifluoroacetic acid.

#### LC RUN (140 min)

Peptides were separated during a biphasic ACN gradient from two nanoflow UPLC pumps (flow rate of 250 nL/min) on a 25 cm analytical column (PepMap RSLC, 25 cm x 75 μm ID. EASY-spray column, packed with 2 μm C18 beads). Solvent A and B were 0.1% FA (vol/vol) in water and 100% ACN, respectively. The gradient composition was 5%B during trapping (5 min) followed by 5–7%B over 1 min, 7–22%B for the next 84 min, 22–28%B over 10 min, and 28–85%B over 5 min. Elution of very hydrophobic peptides and conditioning of the column were performed for 15 min isocratic elution with 85%B and 15 min isocratic conditioning with 5%B, respectively. Instrument control was through Thermo Scientific SII for Xcalibur 1.6.

#### High field asymmetric waveform ion mobility spectrometry (FAIMS)

The FAIMS filter performs gas-phase fractionation, enabling preferred accumulation of multiply charged ions to maximize the efficiency of data-dependent acquisition (DDA). FAIMS results in less precursor co-isolation (improved MS2 TMT quantification), and cleaner MS2 spectra. Short-ion residence time in the FAIMS Pro interface electrode assembly enables use of multiple CV settings in a single run to increase proteome coverage.

#### DDA with FAIMS

Peptides eluted from the column were detected in the Orbitrap Eclipse Mass Spectrometer with FAIMS enabled using two compensation voltages (CVs), −50 V, and −70. During each CV, the mass spectrometer was operated in the DDA-mode (data-dependent-acquisition) to automatically switch between one full scan MS and MS/MS acquisition. Instrument control was through Orbitrap Eclipse Tune 3.5 and Xcalibur 4.5. The cycle time was maintained at 1.5 s/CV. MS spectra were acquired in the scan range 375–1,500 m/z with resolution *R* = 120,000 at m/z 200, automatic gain control (AGC) target of 4e5 and a maximum injection time (IT) set to Auto. The most intense eluting peptides with charge states 2 to 6 were sequentially isolated to a target value (AGC) of 5e4 and a maximum IT of 75 ms in the C-trap, and isolation width maintained at 1.6 m/z (quadrupole isolation), before fragmentation in the HCD (Higher-Energy Collision Dissociation). Fragmentation was performed with a normalized collision energy (NCE) of 32%, and fragments were detected in the Orbitrap at a resolution of 50,000 at m/z 200, with first mass fixed at m/z 110. One MS/MS spectrum of a precursor mass was allowed before dynamic exclusion for 30s with “exclude isotopes” on. Lock-mass internal calibration was not enabled.

#### Ion source parameter

The spray and ion-source parameters were as follows. Ion spray voltage = 2000 V, no sheath and auxiliary gas flow, and capillary temperature = 275 °C.

### Data analysis

#### Spheroid data

For each cell line and treatment condition, 46 spheroids were analyzed using the Brightfield channel in the IncuCyte Spheroid Analysis Software Module to identify spheroid boundaries and quantification of spheroid area.

#### Spheroid invasion assay

To measure breast cancer spheroid cell invasion into surrounding extracellular matrix (Matrigel, Corning #356234), we generated spheroids by seeding MDA-MB-231 cells (5,000 cells per well), in a ULA round bottom 96-well plate (Corning #7007). Spheroids were allowed to form for 3 days in an incubator at 37 °C, 5% CO_2_. Spheroids were subsequently embedded in Matrigel (4.45 mg/mL) on day 3 and the different treatments were added (noradrenaline 10 μM, propranolol 10 μM). IncuCyte depth of focus brightfield (DF-BF) images were obtained every 6 h for 4 consecutive days allowing us to quantify invading cells and whole spheroid area. Time course plots of Invading Cell BF Area were generated to show differences in invasive capacity of breast cancer cells in the presence or absence of noradrenaline and propranolol.

#### Proteomics data

Heatmap, GSEA and UMAP (Uniform Manifold Approximation and Projection) visualizations were generated using RStudio. For UMAP, GSEA and heatmap, the entire proteome was analyzed both pre- and post-drug treatment. Data was imported and processed in R using in-house generated scripts.

Gene enrichment analysis was conducted to study the abundance of protein values and their biological function (Liberzon et al., 2015; Subramanian et al., 2005). The molecular signature database (MSigDB) was utilized for its collection of annotated gene sets. Where we utilized the H hallmark gene sets and the C6 oncogenic signature gene sets to determine enrichment. Gene sets criteria were minimum 20 and maximum 1,000 per term, while Benjamini-Hockberg adjusted *p*-value cutoff was set at 0.05. Log2 fold change between the drug treatment and control was utilized as the input data. Upregulated gene sets were determined as normalized enrichment score (NES) > 0, while downregulated were determined as < 0.

Proteins significant by an alpha level of 0.05 were uploaded to Pathway Ingenuity Pathway Analysis program (IPA®, QIAGEN Redwood City, www.qiagen.com/ingenuity), working as previously described (Krämer et al., 2014). In brief, following settings were used for individual analyses: Expression Fold Change (Exp Fold Change), Relationships to consider (Direct and Indirect Relationships), Reference set (Corresponding data analysis), Interaction networks (35 molecules/network; 25 networks/analysis), Species (mammal: Human, mouse, rat), Molecule & Canonical Pathway subcategories were determined by “all” data types if not otherwise stated. Comparison pathway analysis was performed in IPA based on the individual datasets analyses.

#### Transcriptomic expression data

Transcriptomic expression data for ADRB2 were retrieved from the Cancer Cell Line Encyclopedia (CCLE) via the DepMap portal.^1^ mRNA expression values (log₂ [TPM + 1]) from the Public 25Q2 RNA-seq dataset were extracted for MDA-MB-231, BT549, MCF7, and T47D cell lines. Data were visualized using Prism (GraphPad Software).

## Data Availability

The datasets generated for this study can be found in the ProteomeXchange Consortium via the PRIDE repository with the dataset identifier PXD046841. The underlying codes and programs implemented for this study may be available upon reasonable request to the corresponding author.

## Footnotes

^1^ https://depmap.org/portal/

## References

[ref1] Amaro F. Silva D. Reguengo H. Oliveira J. C. Quintas C. Vale N.. (2020). β-Adrenoceptor activation in breast MCF-10A cells induces a pattern of catecholamine production similar to that of tumorigenic MCF-7 cells. Int. J. Mol. Sci. 21:7968. doi: 10.3390/ijms21217968, PMID: 33120955 PMC7672553

[ref2] Baker J. G. Hill S. J. Summers R. J. (2011). Evolution of beta-blockers: from anti-anginal drugs to ligand-directed signalling. Trends Pharmacol. Sci. 32, 227–234. doi: 10.1016/j.tips.2011.02.010 21429598 PMC3081074

[ref3] Barron T. I. Connolly R. M. Sharp L. Bennett K. Visvanathan K. (2011). Beta blockers and breast cancer mortality: a population- based study. J. Clin. Oncol. 29, 2635–2644. doi: 10.1200/JCO.2010.33.5422 21632503

[ref4] Beg M. S. Gupta A. Sher D. Ali S. Khan S. Gao A.. (2018). Impact of concurrent medication use on pancreatic Cancer survival-SEER-Medicare analysis. Am. J. Clin. Oncol. 41, 766–771. doi: 10.1097/COC.0000000000000359, PMID: 28079594 PMC5503814

[ref5] Bjornstad O. V. Carrasco M. Finne K. Ardawatia V. Winge I. Askeland C.. (2024). Global and single-cell proteomics view of the co-evolution between neural progenitors and breast cancer cells in a co-culture model. EBioMedicine 108:105325. doi: 10.1016/j.ebiom.2024.105325, PMID: 39232464 PMC11404160

[ref6] Cardwell C. R. Coleman H. G. Murray L. J. Entschladen F. Powe D. G. (2013). Beta-blocker usage and breast cancer survival: a nested case-control study within a UK clinical practice research datalink cohort. Int. J. Epidemiol. 42, 1852–1861. doi: 10.1093/ije/dyt196, PMID: 24536096

[ref7] Carracedo A. Cantley L. C. Pandolfi P. P. (2013). Cancer metabolism: fatty acid oxidation in the limelight. Nat. Rev. Cancer 13, 227–232. doi: 10.1038/nrc3483, PMID: 23446547 PMC3766957

[ref8] Carrasco M. Wang C. Søviknes A. M. Bjørlykke Y. Abadpour S. Paulo J. A.. (2022). Spatial environment affects HNF4A mutation-specific proteome signatures and cellular morphology in hiPSC-derived β-like cells. Diabetes 71, 862–869. doi: 10.2337/db20-1279, PMID: 35043148 PMC8965667

[ref9] Choi M. J. Cho K. H. Lee S. Bae Y. J. Jeong K. J. Rha S. Y.. (2015). hTERT mediates norepinephrine-induced slug expression and ovarian cancer aggressiveness. Oncogene 34, 3402–3412. doi: 10.1038/onc.2014.270, PMID: 25151968

[ref10] Eng J. W. Kokolus K. M. Reed C. B. Hylander B. L. Ma W. W. Repasky E. A. (2014). A nervous tumor microenvironment: the impact of adrenergic stress on cancer cells, immunosuppression, and immunotherapeutic response. Cancer Immunol. Immunother. 63, 1115–1128. doi: 10.1007/s00262-014-1617-9, PMID: 25307152 PMC4325998

[ref11] Gargiulo L. Rivero E. M. di Siervi N. Buzzi E. D. Buffone M. G. Davio C. A.. (2020). Agonist effects of propranolol on non-tumor human breast cells. Cells 9:1036. doi: 10.3390/cells9041036, PMID: 32331276 PMC7226086

[ref12] German N. J. Haigis M. C. (2015). Sirtuins and the metabolic hurdles in Cancer. Curr. Biol. 25, R569–R583. doi: 10.1016/j.cub.2015.05.012, PMID: 26126285 PMC5748939

[ref13] Jabloñski M. Rodríguez M. S. Rivero E. M. Bruque C. D. Vanzulli S. Bruzzone A.. (2023). The Beta2-adrenergic agonist salbutamol synergizes with paclitaxel on cell proliferation and tumor growth in triple negative breast cancer models. Cancer Chemother. Pharmacol. 92, 485–499. doi: 10.1007/s00280-023-04586-9, PMID: 37725114

[ref14] Jansen L. Hoffmeister M. Arndt V. Chang-Claude J. Brenner H. (2014). Stage-specific associations between beta blocker use and prognosis after colorectal cancer. Cancer 120, 1178–1186. doi: 10.1002/cncr.28546, PMID: 24415516

[ref15] Jayachandran P. Battaglin F. Strelez C. Lenz A. Algaze S. Soni S.. (2023). Breast cancer and neurotransmitters: emerging insights on mechanisms and therapeutic directions. Oncogene 42, 627–637. doi: 10.1038/s41388-022-02584-4, PMID: 36650218 PMC9957733

[ref16] Jin M. Wang Y. Zhou T. Li W. Wen Q. (2023). Norepinephrine/β(2)-adrenergic receptor pathway promotes the cell proliferation and nerve growth factor production in triple-negative breast Cancer. J. Breast Cancer 26, 268–285. doi: 10.4048/jbc.2023.26.e25, PMID: 37387350 PMC10315331

[ref17] Karemaker J. M. (2017). An introduction into autonomic nervous function. Physiol. Meas. 38, R89–r118. doi: 10.1088/1361-6579/aa6782, PMID: 28304283

[ref18] Kenny P. A. Lee G. Y. Myers C. A. Neve R. M. Semeiks J. R. Spellman P. T.. (2007). The morphologies of breast cancer cell lines in three-dimensional assays correlate with their profiles of gene expression. Mol. Oncol. 1, 84–96. doi: 10.1016/j.molonc.2007.02.004, PMID: 18516279 PMC2391005

[ref19] Kim H. Y. Jung Y. J. Lee S. H. Jung H. J. Pak K. (2017). Is Beta-blocker use beneficial in breast Cancer? A Meta-Analysis. Oncology 92, 264–268. doi: 10.1159/000455143, PMID: 28132057

[ref20] Kim-Fuchs C. Le C. P. Pimentel M. A. Shackleford D. Ferrari D. Angst E.. (2014). Chronic stress accelerates pancreatic cancer growth and invasion: a critical role for beta-adrenergic signaling in the pancreatic microenvironment. Brain Behav. Immun. 40, 40–47. doi: 10.1016/j.bbi.2014.02.019, PMID: 24650449 PMC4102665

[ref21] Krämer A. Green J. Pollard J. Jr. Tugendreich S. (2014). Causal analysis approaches in ingenuity pathway analysis. Bioinformatics 30, 523–530. doi: 10.1093/bioinformatics/btt703, PMID: 24336805 PMC3928520

[ref22] Leroy B. Girard L. Hollestelle A. Minna J. D. Gazdar A. F. Soussi T. (2014). Analysis of TP53 mutation status in human cancer cell lines: a reassessment. Hum. Mutat. 35, 756–765. doi: 10.1002/humu.22556, PMID: 24700732 PMC4451114

[ref23] Liberzon A. Birger C. Thorvaldsdóttir H. Ghandi M. Mesirov Jill P. Tamayo P. (2015). The molecular signatures database Hallmark gene set collection. Cell Systems 1, 417–425. doi: 10.1016/j.cels.2015.12.004, PMID: 26771021 PMC4707969

[ref24] Lofling L. L. Stoer N. C. Sloan E. K. Chang A. Gandini S. Ursin G.. (2022). beta-blockers and breast cancer survival by molecular subtypes: a population-based cohort study and meta-analysis. Br. J. Cancer 127, 1086–1096. doi: 10.1038/s41416-022-01891-7, PMID: 35725814 PMC9470740

[ref25] Lorona N. C. Cook L. S. Tang M. C. Hill D. A. Wiggins C. L. Li C. I. (2021). Antihypertensive medications and risks of recurrence and mortality in luminal, triple-negative, and HER2-overexpressing breast cancer. Cancer Causes Control 32, 1375–1384. doi: 10.1007/s10552-021-01485-3, PMID: 34347212 PMC8541909

[ref26] Manuel Iglesias J. Beloqui I. Garcia-Garcia F. Leis O. Vazquez-Martin A. Eguiara A.. (2013). Mammosphere formation in breast carcinoma cell lines depends upon expression of E-cadherin. PLoS One 8:e77281. doi: 10.1371/journal.pone.0077281, PMID: 24124614 PMC3790762

[ref27] Melhem-Bertrandt A. Chavez-Macgregor M. Lei X. Brown E. N. Lee R. T. Meric-Bernstam F.. (2011). Beta-blocker use is associated with improved relapse-free survival in patients with triple-negative breast cancer. J. Clin. Oncol. 29, 2645–2652. doi: 10.1200/JCO.2010.33.4441 21632501 PMC3139371

[ref28] Modi N. D. Tan J. Q. E. Rowland A. Koczwara B. Kichenadasse G. McKinnon R. A.. (2020). The influence of pre-existing beta-blockers use on survival outcomes in HER2 positive advanced breast cancer: pooled analysis of clinical trial data. Front. Oncol. 10:1130. doi: 10.3389/fonc.2020.01130, PMID: 32760671 PMC7373122

[ref29] Nilsson M. B. Armaiz-Pena G. Takahashi R. Lin Y. G. Trevino J. Li Y.. (2007). Stress hormones regulate interleukin-6 expression by human ovarian carcinoma cells through a Src-dependent mechanism. J. Biol. Chem. 282, 29919–29926. doi: 10.1074/jbc.M611539200, PMID: 17716980

[ref30] Nolan E. Lindeman G. J. Visvader J. E. (2023). Deciphering breast cancer: from biology to the clinic. Cell 186, 1708–1728. doi: 10.1016/j.cell.2023.01.040, PMID: 36931265

[ref31] Ouyang X. Zhu Z. Yang C. Wang L. Ding G. Jiang F. (2019). Epinephrine increases malignancy of breast cancer through p38 MAPK signaling pathway in depressive disorders. Int. J. Clin. Exp. Pathol. 12, 1932–1946. 31934016 PMC6949628

[ref32] Overman J. Fontaine F. Wylie-Sears J. Moustaqil M. Huang L. Meurer M.. (2019). R-propranolol is a small molecule inhibitor of the SOX18 transcription factor in a rare vascular syndrome and hemangioma. eLife 8:e43026. doi: 10.7554/eLife.43026, PMID: 31358114 PMC6667216

[ref33] Pasquier E. Ciccolini J. Carre M. Giacometti S. Fanciullino R. Pouchy C.. (2011). Propranolol potentiates the anti-angiogenic effects and anti-tumor efficacy of chemotherapy agents: implication in breast cancer treatment. Oncotarget 2, 797–809. doi: 10.18632/oncotarget.343, PMID: 22006582 PMC3248157

[ref34] Renz B. W. Takahashi R. Tanaka T. Macchini M. Hayakawa Y. Dantes Z.. (2018). β2 adrenergic-Neurotrophin feedforward loop promotes pancreatic Cancer. Cancer Cell 33, 75–90.e7. doi: 10.1016/j.ccell.2017.11.007, PMID: 29249692 PMC5760435

[ref35] Rivero E. M. Piñero C. P. Gargiulo L. Entschladen F. Zänker K. Bruzzone A.. (2017). The β 2-adrenergic agonist salbutamol inhibits migration, invasion and metastasis of the human breast cancer MDA-MB- 231 cell line. Curr. Cancer Drug Targets 17, 756–766. doi: 10.2174/1568009617666170330151415, PMID: 28359245

[ref36] Saraiva D. P. Matias A. T. Braga S. Jacinto A. Cabral M. G. (2020). Establishment of a 3D co-culture with MDA-MB-231 breast cancer cell line and patient-derived immune cells for application in the development of immunotherapies. Front. Oncol. 10, 1–13. doi: 10.3389/fonc.2020.01543 32974189 PMC7482668

[ref37] Scott O. W. Tin Tin S. Elwood J. M. Cavadino A. Habel L. A. Kuper-Hommel M.. (2022). Post-diagnostic beta blocker use and breast cancer-specific mortality: a population-based cohort study. Breast Cancer Res. Treat. 193, 225–235. doi: 10.1007/s10549-022-06528-0, PMID: 35286523 PMC8993732

[ref38] Scott O. W. TinTin S. Cavadino A. Elwood J. M. (2024). Beta-blocker use and breast cancer outcomes: a meta-analysis. Breast Cancer Res. Treat. 206, 443–463. doi: 10.1007/s10549-024-07263-4, PMID: 38837086 PMC11208256

[ref39] Silva D. Quintas C. Gonçalves J. Fresco P. (2024). β2-adrenoceptor activation favor Acquisition of Tumorigenic Properties in non-tumorigenic MCF-10A breast epithelial cells. Cells 13:262. doi: 10.3390/cells13030262, PMID: 38334654 PMC10854540

[ref40] Sørensen G. V. Ganz P. A. Cole S. W. Pedersen L. A. Sørensen H. T. Cronin-Fenton D. P.. (2013). Use of β-blockers, angiotensin-converting enzyme inhibitors, angiotensin II receptor blockers, and risk of breast cancer recurrence: a Danish nationwide prospective cohort study. J. Clin. Oncol. 31, 2265–2272. doi: 10.1200/JCO.2012.43.9190, PMID: 23650417 PMC3677839

[ref41] Spera G. Fresco R. Fung H. Dyck J. R. B. Pituskin E. Paterson I.. (2017). Beta blockers and improved progression-free survival in patients with advanced HER2 negative breast cancer: a retrospective analysis of the ROSE/TRIO-012 study. Ann. Oncol. 28, 1836–1841. doi: 10.1093/annonc/mdx264, PMID: 28520849

[ref42] Subramanian A. Tamayo P. Mootha V. K. Mukherjee S. Ebert B. L. Gillette M. A.. (2005). Gene set enrichment analysis: a knowledge-based approach for interpreting genome-wide expression profiles. Proc. Natl. Acad. Sci. USA 102, 15545–15550. doi: 10.1073/pnas.0506580102, PMID: 16199517 PMC1239896

[ref43] Sung H. Ferlay J. Siegel R. L. Laversanne M. Soerjomataram I. Jemal A.. (2021). Global cancer statistics 2020: GLOBOCAN estimates of incidence and mortality worldwide for 36 cancers in 185 countries. CA Cancer J. Clin. 71, 209–249. doi: 10.3322/caac.21660, PMID: 33538338

[ref44] Watkins J. L. Thaker P. H. Nick A. M. Ramondetta L. M. Kumar S. Urbauer D. L.. (2015). Clinical impact of selective and nonselective beta-blockers on survival in patients with ovarian cancer. Cancer 121, 3444–3451. doi: 10.1002/cncr.29392, PMID: 26301456 PMC4575637

[ref45] Wehrwein E. A. Orer H. S. Barman S. M. (2016). Overview of the anatomy, physiology, and pharmacology of the autonomic nervous system. Compr. Physiol. 6, 1239–1278. doi: 10.1002/j.2040-4603.2016.tb00714.x, PMID: 27347892

[ref46] Winter M. Meignan S. Volkel P. Angrand P. O. Chopin V. Bidan N.. (2021). Vimentin promotes the aggressiveness of triple negative breast Cancer cells surviving chemotherapeutic treatment. Cells 10:1504. doi: 10.3390/cells10061504, PMID: 34203746 PMC8232646

[ref47] Yersal O. Barutca S. (2014). Biological subtypes of breast cancer: prognostic and therapeutic implications. World J Clin Oncol. 5, 412–424. doi: 10.5306/wjco.v5.i3.412, PMID: 25114856 PMC4127612

[ref48] Yue J. Zhang K. Chen J. (2012). Role of integrins in regulating proteases to mediate extracellular matrix remodeling. Cancer Microenviron. 5, 275–283. doi: 10.1007/s12307-012-0101-3, PMID: 22437309 PMC3460049

[ref49] Zahalka A. H. Arnal-Estape A. Maryanovich M. Nakahara F. Cruz C. D. Finley L. W. S.. (2017). Adrenergic nerves activate an angio-metabolic switch in prostate cancer. Science 358, 321–326. doi: 10.1126/science.aah5072, PMID: 29051371 PMC5783182

[ref50] Zahalka A. H. Frenette P. S. (2020). Nerves in cancer. Nat. Rev. Cancer 20, 143–157. doi: 10.1038/s41568-019-0237-2, PMID: 31974491 PMC7709871

[ref51] Zhang D. Ma Q. Y. Hu H. T. Zhang M. (2010). Β2-adrenergic antagonists suppress pancreatic cancer cell invasion by inhibiting CREB, NFκB and AP-1. Cancer Biol. Ther. 10, 19–29. doi: 10.4161/cbt.10.1.11944, PMID: 20424515

[ref52] Zhang B. Wu C. Chen W. Qiu L. Li S. Wang T.. (2020). The stress hormone norepinephrine promotes tumor progression through β2-adrenoreceptors in oral cancer. Arch. Oral Biol. 113:104712. doi: 10.1016/j.archoralbio.2020.104712, PMID: 32234582

