# Supplementary material for: Adrenergic signals influence proteomic responses in breast cancer cells
Source: Front Neurosci. 2025 Sep 17;19:1608017. doi: 10.3389/fnins.2025.1608017 (PMC12483918; doi:10.3389/fnins.2025.1608017)
Supplement: Supplementary file 1 [file Data_Sheet_1.DOCX]

**Supplementary Tables**

| **Whole spheroid area** | |  |  |  |
| --- | --- | --- | --- | --- |
|  | **Ctrl** | **N** | **P** | **P + N** |
| Total Area | 31768546 | 17099194 | 34024417 | 25929146 |
| Std. Error | 548174 | 905057 | 965610 | 859886 |
| 95% Confidence Interval | 30694144 to 32842947 | 15325315 to 18873073 | 32131857 to 35916978 | 24243800 to 27614492 |
|  |  |  |  |  |
| **Invading cells area** | |  |  |  |
|  | **Ctrl** | **N** | **P** | **P + N** |
| Total Area | 13565780 | 8087109 | 13499369 | 8370311 |
| Std. Error | 564076 | 583578 | 886482 | 465476 |
| 95% Confidence Interval | 12460211 to 14671348 | 6943317 to  9230901 | 11761896 to 15236842 | 7457996 to  9282627 |

**Supplementary Table 1.** Area under the curve (AUC) values and 95% confidence intervals (CI) for whole spheroid area and invading cell areas in MDA-MB-231 spheroids under different conditions.

**Supplementary Figures**


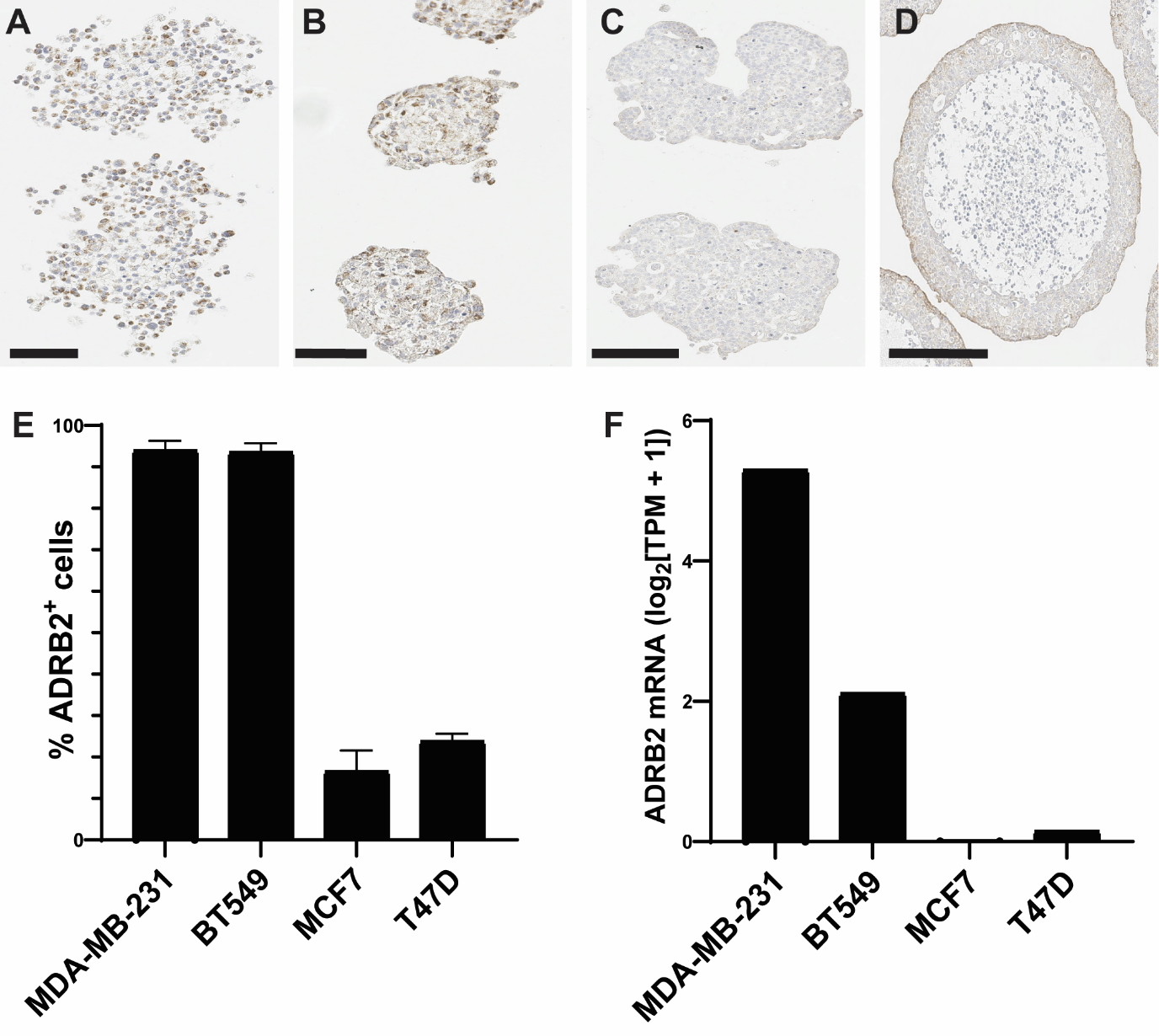


**Supplementary Figure 1. β2-Adrenergic receptor (ADRB2) immunohistochemistry in breast cancer spheroids.** Representative sections of spheroids derived from MDA-MB-231 (**A**), BT549 (**B**), MCF7 (**C**), and T47D (**D**) stained for by IHC. Strong staining was observed in MDA-MB-231 and BT549 cells, while luminal-like MCF7 and T47D spheroids showed minimal ADRB2 immunoreactivity. **(E)** Quantification of ADRB2-positive cells (% positive cells). **(F)** mRNA expression levels of ADRB2 (log₂[TPM+1]) retrieved from the Cancer Cell Line Encyclopedia (CCLE) RNA-seq dataset (DepMap Public 25Q2 release). Scale bars = 100 µm.


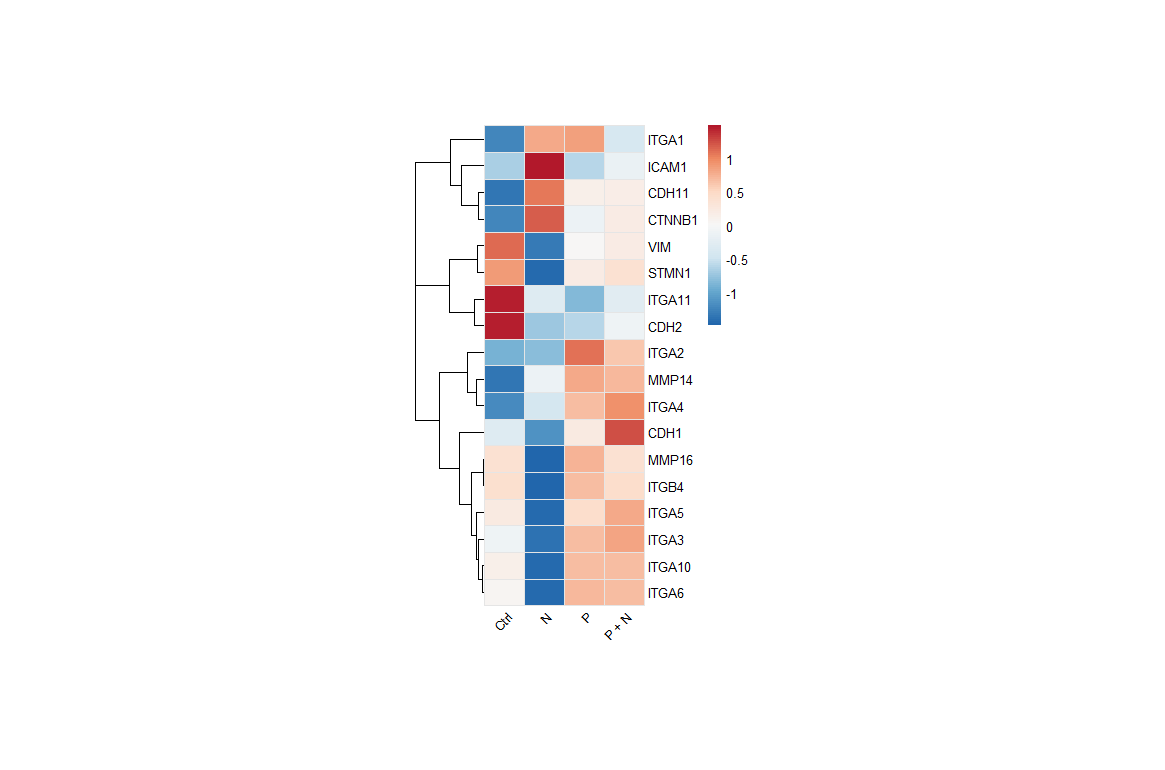


**Supplementary Figure 2: Expression of EMT-related markers and integrin subunits in MDA-MB-231 spheroids following adrenergic stimulation.** Heatmap representing z-score normalized protein expression levels for Vimentin (VIM), Stathmin 1 (STMN1), matrix metalloproteinases (MMP14, MMP16), cadherins (CDH1, CDH2, CDH11), β-catenin (CTNNB1), integrin subunits (ITGA1, ITGA2, ITGA3, ITGA4, ITGA5, ITGA6, ITGA10, ITGA11, ITGB4), and ICAM1 in spheroids under four conditions: untreated control (Ctrl), noradrenaline (N, 10 µM), propranolol (P, 10 µM), and combined treatment (P + N).


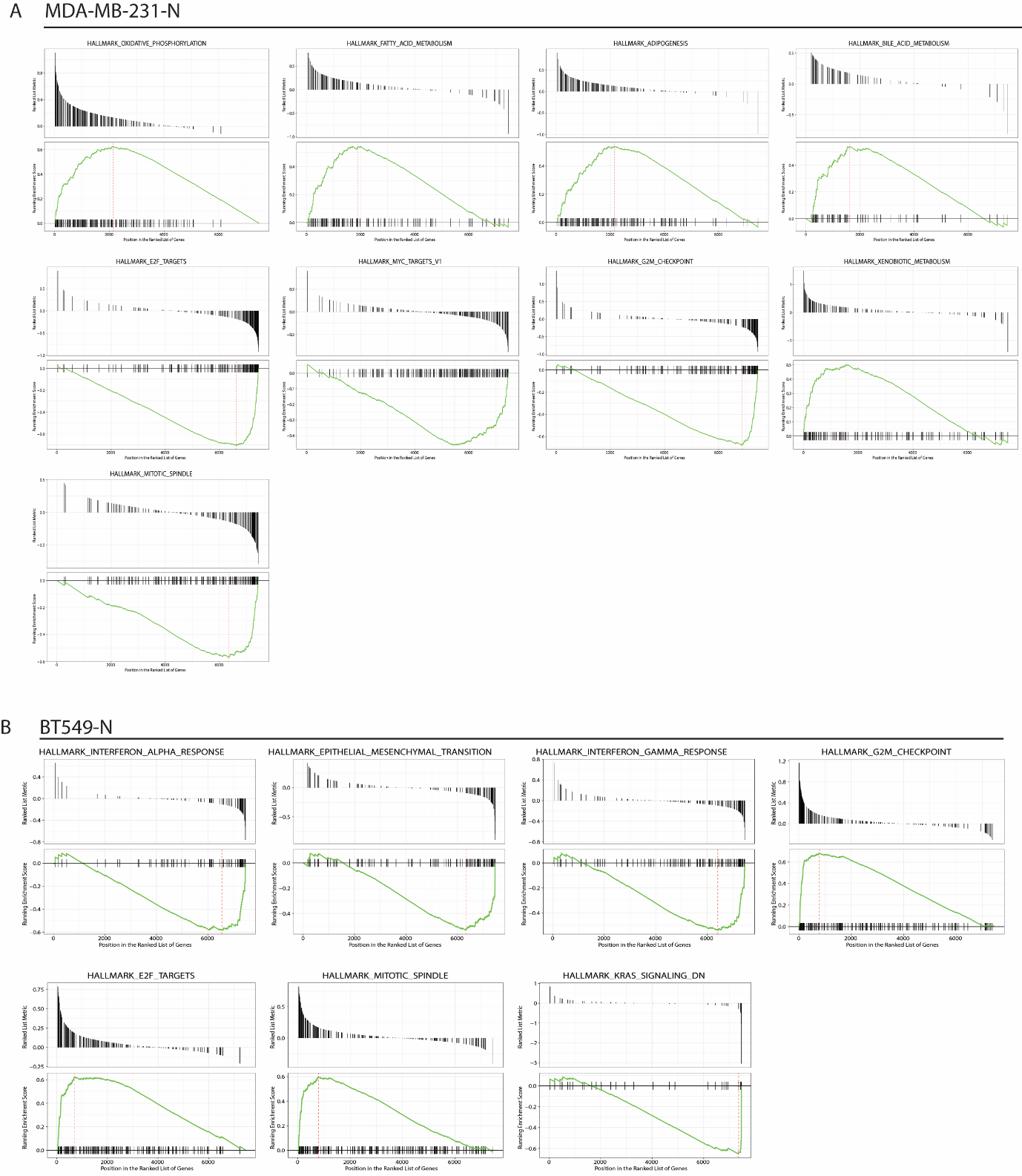


**Supplementary Figure 3: Gene Set Enrichment Analysis (GSEA) comparisons for MDA-MB-231 and BT549 cell lines with noradrenalin treatment.** This figure presents the GSEA enrichment plots for (**A**) MDA-MB-231-N and (**B**) BT549-N, each treated with noradrenalin, compared to their respective untreated controls, showing statistically significant (p < 0.05) gene sets within the "hallmark gene sets" category from the human Molecular Signatures Database (MSigDB).


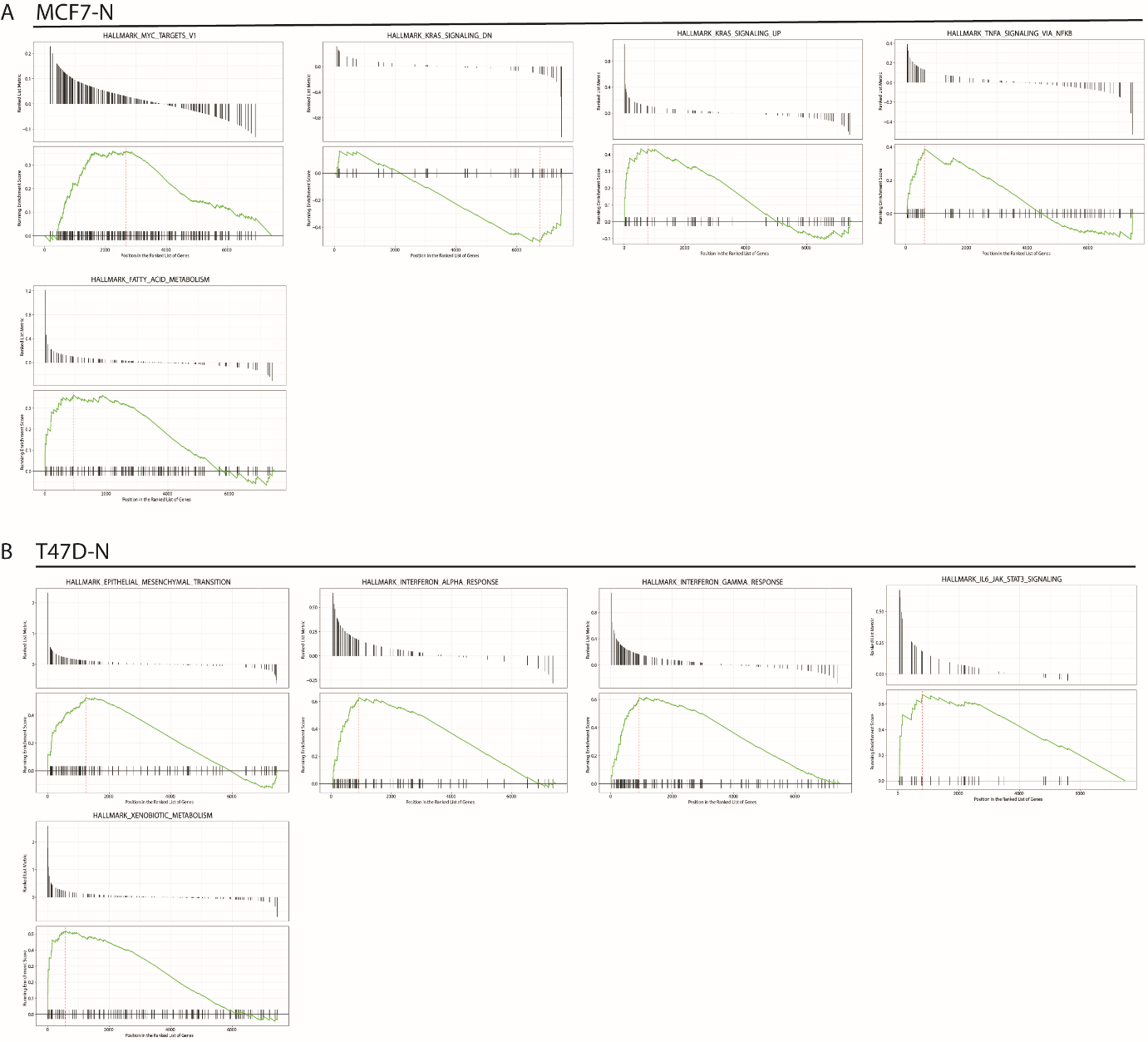


**Supplementary Figure 4: Gene Set Enrichment Analysis (GSEA) comparisons for MCF7 and T47D cell lines with noradrenalin treatment.** Showing the GSEA enrichment plots for (**A**) MCF7 and (**B**) T47D, each treated with noradrenalin, compared to their respective untreated controls, displaying statistically significant (p < 0.05) gene sets within the "hallmark gene sets" category from the human Molecular Signatures Database (MSigDB).
